# Spatiotemporal Neural Network for Sublexical Information Processing: An Intracranial SEEG Study

**DOI:** 10.1523/JNEUROSCI.0717-24.2024

**Published:** 2024-08-30

**Authors:** Chunyu Zhao, Yi Liu, Jiahong Zeng, Xiangqi Luo, Weijin Sun, Guoming Luan, Yuxin Liu, Yumei Zhang, Gaofeng Shi, Yuguang Guan, Zaizhu Han

**Affiliations:** ^1^National Key Laboratory of Cognitive Neuroscience and Learning, Beijing Normal University, Beijing 100875, China; ^2^Beijing Institute of Otolaryngology, Otolaryngology-Head and Neck Surgery, Beijing Tongren Hospital, Capital Medical University, Beijing 100005, China; ^3^Department of Neurosurgery, Sanbo Brain Hospital, Capital Medical University, Beijing 100093, China; ^4^Department of Rehabilitation Medicine, Beijing Tiantan Hospital, Capital Medical University, Beijing 100070, China; ^5^College of International Education and Exchange, Tianjin Normal University, Tianjin 300387, China

**Keywords:** SEEG, spatiotemporal network, sublexical processing, word reading

## Abstract

Words offer a unique opportunity to separate the processing mechanisms of object subcomponents from those of the whole object, because the phonological or semantic information provided by the word subcomponents (i.e., sublexical information) can conflict with that provided by the whole word (i.e., lexical information). Previous studies have revealed some of the specific brain regions and temporal information involved in sublexical information processing. However, a comprehensive spatiotemporal neural network for sublexical processing remains to be fully elucidated due to the low temporal or spatial resolutions of previous neuroimaging studies. In this study, we recorded stereoelectroencephalography signals with high spatial and temporal resolutions from a large sample of 39 epilepsy patients (both sexes) during a Chinese character oral reading task. We explored the activated brain regions and their connectivity related to three sublexical effects: phonological regularity (whether the whole character's pronunciation aligns with its phonetic radical), phonological consistency (whether characters with the same phonetic radical share the same pronunciation), and semantic transparency (whether the whole character's meaning aligns with its semantic radical). The results revealed that sublexical effects existed in the inferior frontal gyrus, precentral and postcentral gyri, temporal lobe, and middle occipital gyrus. Additionally, connectivity from the middle occipital gyrus to the postcentral gyrus and from postcentral gyrus to the fusiform gyrus was associated with the sublexical effects. These findings provide valuable insights into the spatiotemporal dynamics of sublexical processing and object recognition in the brain.

## Significance Statement

Elucidating the intricate neural mechanisms underlying sublexical processing is crucial for understanding the intricacies of language comprehension and object recognition in the human brain. This study employed intracranial stereoelectroencephalography recordings to investigate the spatiotemporal dynamics of sublexical processing during a Chinese character reading task. We constructed a neural network for sublexical processing and depicted its temporal sequence in different brain regions. Furthermore, we identified the information flow within this network and observed its variation with the reading of characters containing different sublexical information. These findings not only advance our understanding of the cerebral mechanisms governing sublexical processing but also offer insights into the broader framework of object recognition processes.

## Introduction

Rapid and accurate object recognition is crucial for creatures’ survival (Logothetis and Sheinberg, 1996). Recognition aims to identify the entire object, but efficient recognition often requires considering subcomponents (Ullman, 2007), as the Chinese idiom goes, “To observe a spot and know the leopard.” Thus, the processing of subcomponents is a significant and relevant scientific issue (Biederman, 1987). However, distinguishing the processing mechanisms of subcomponents from those of whole objects is challenging, as both whole objects and their components often provide consistent information in the natural world (Hoffman and Richards, 1984). Fortunately, there is an exception: human-invented words, wherein the phonological or semantic information provided by the subcomponents (sublexical information) can conflict with that of the whole word (lexical information; Zhou and Marslen-Wilson, 1999; Kim et al., 2004; Mok, 2009). For example, the pronunciation of the English word “business” cannot be predicted based on its sublexical component “bus” (Libben et al., 2003; Borgwaldt et al., 2005). Therefore, words offer a unique opportunity to reveal the processing of object subcomponents.

Approximately 81% of Chinese characters are composited, comprising a phonetic and semantic radical (sublexical components specific to Chinese; Li and Kang, 1993). These radicals provide congruent or incongruent information to the whole character (Xing, 2006). Based on limited studies (Yum et al., 2014; Wang et al., 2016; Dang et al., 2019), researchers have examined sublexical processing through phonological consistency, phonological regularity, and semantic transparency effects. For example, “清” [/*qing1*/ (pronunciation), limpid (meaning)] has a phonetic radical “青” (/*qing1*/) and a semantic radical “氵” (water), both aligning with the character's pronunciation and meaning, making it phonologically regular and semantically transparent. Conversely, “猜” (/*cai2*/, guess) has a phonetic radical “青” and a semantic radical “犭” (animal), neither of which align with the character's pronunciation and meaning, making it phonologically irregular and semantically opaque. The consistency effect refers to the uniformity of pronunciations among all characters sharing the same phonetic radical. For instance, characters (e.g., 蝗, 惶, 煌, 蝗) sharing the phonetic radical are all pronounced as /*huang2*/, ensuring consistency in pronunciation. Conversely, 清 (/*qing1*/), 猜 (/*cai1*/), 倩 (/*qian4*/), and 精 (/*jing1*/) share the phonetic radical but have different pronunciations, indicating inconsistency. Therefore, regularity refers to whether the pronunciation of the character is the same as its phonetic radical, while consistency refers to whether the pronunciation of the character is the same as that of other family characters. A Chinese character can be regular but inconsistent; however, it cannot be irregular yet consistent. Regularity and consistency effects reflect phonological profiles, while transparency effects represent semantic profiles (Zhou and Marslen-Wilson, 1999).

All the three sublexical effects have been observed in Chinese character recognition (Feldman and Siok, 1999; Wu et al., 1999; Zhou and Marslen-Wilson, 1999; Ding et al., 2004; Tsang and Chen, 2009; Yan et al., 2012; Zhou et al., 2013). For instance, behavioral studies have shown that regular, consistent, and transparent characters are read faster than irregular, inconsistent, and opaque characters, respectively (Hue, 1992; Zhou and Marslen-Wilson, 1999; Ding et al., 2004; Bi et al., 2007; Wang et al., 2016). Functional MRI (fMRI) studies have identified the brain regions that process Chinese character radicals, including the temporoparietal cortex and frontal lobe (Hunag et al., 2010; Yang et al., 2011; Wu et al., 2013; Liu et al., 2022). Event-related potential (ERP) research revealed that sublexical semantic processing occurs as early as 50–100 ms poststimulus in the brain (Lee et al., 2006; Wang et al., 2018), while sublexical phonological processing occurs 170–400 ms (Yum et al., 2014; Zhou et al., 2014).

The aforementioned studies revealed that sublexical components are decomposed and involved in character recognition. The studies also identified specific brain regions and temporal information involved in sublexical processing. However, a comprehensive spatiotemporal neural network of sublexical processing has not been elucidated due to the following reasons. First, to our knowledge, no individual neuroimaging study has examined and compared the sublexical effects of regularity, consistency, and transparency together, which limits our understanding of how neural networks converge and diverge across different types of sublexical processes. Second, due to the limited temporal resolution of fMRI and the low spatial resolution of ERP studies, the precise time course of sublexical information within each sublexical brain region remains unclear. Finally, it is also unclear how each type of sublexical information is transferred between sublexical brain regions.

**Figure 1.**
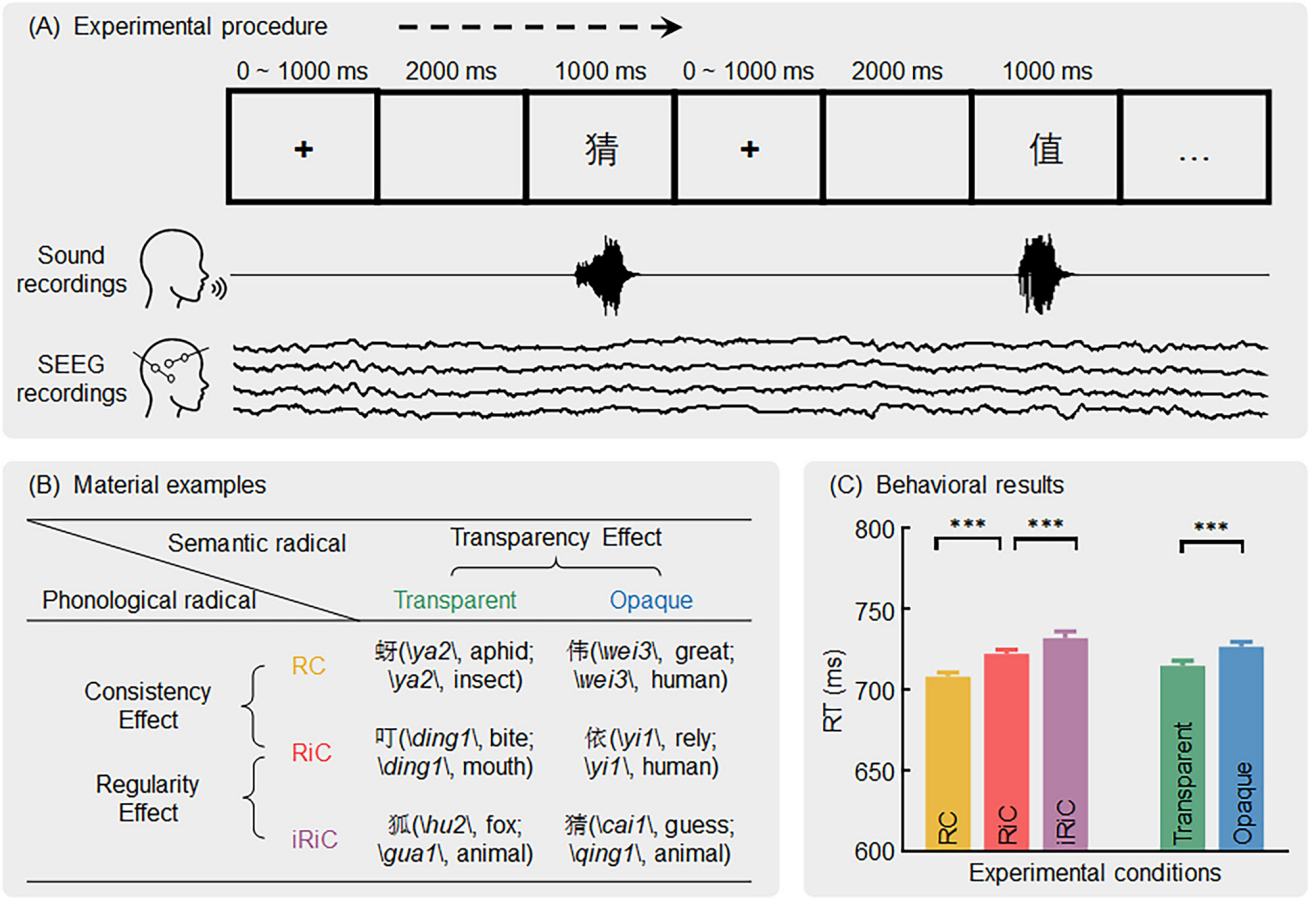
Schematic diagram of the experimental procedure, material examples, and behavioral results. ***A***, Experimental procedure (top) as well as the recordings of sound (middle) and SEEG (bottom). Participants were tasked with reading the stimuli aloud as quickly as possible while maintaining accuracy when the reading materials appeared on the screen. In the sound recordings, the amplitude represents the sound wave. In the SEEG recordings, the amplitude represents the electrophysiological signals captured by multiple contacts in the brain. ***B***, Examples of experimental materials. The first pronunciation and meaning pair explain the pronunciation and meaning of the whole character. The second pair explains the pronunciation of the phonological radical and the meaning of the semantic radical. The consistency effect was assessed by RC versus RiC, the regularity effect was assessed by RiC versus iRiC, and the transparency effect was assessed by transparent versus opaque. ***C***, Behavioral results of RT under different conditions. The trends were as follows: RC < RiC < iRiC, and transparent < opaque, with significant differences between conditions. The error bars represent the standard error. RC, regular-consistent; RiC, regular-inconsistent; iRiC, irregular-inconsistent; RT, response time; ***p < 0.001.

To address these challenges, we recruited 39 epilepsy patients. The participants were instructed to read Chinese characters while undergoing stereoelectroencephalography (SEEG) recordings, which provide precise spatial and temporal resolutions at the millimeter and millisecond levels. These characters were designated to investigate the three sublexical effects (Fig. 1*B*). For each sublexical effect, we identified the relevant regions throughout the entire brain, described the information flow (connectivity) between the regions, and explored behavior-related connections within the network.

## Materials and Methods

### Participants

Thirty-nine epilepsy patients (11 females) were recruited from Sanbo Brain Hospital, Capital Medical University, China. The participants were native Chinese Mandarin speakers, and the majority (34) of them were right-handed (Oldfield, 1971). The mean age was 24.36 years [standard deviation (SD) = 8.03; range, 12–43], with the level of average education being 12.15 years (SD = 3.24; range, 5–17). The patients underwent stereotactic implantation of depth electrodes to locate the seizure zone for clinical treatment. The majority (29) of participants were also included in our recent study (Liu et al., 2021), with 10 new participants in the current study. Informed written consent was obtained from all participants, and the study was approved by the Institutional Review Board of the National Key Laboratory of Cognitive Neuroscience and Learning at Beijing Normal University.

### Experimental materials and design

In contrast to our recent study (Liu et al., 2021) using a lexical decision task, the current study employed an oral reading task. Participants were tasked with reading the stimuli aloud as quickly as possible while maintaining accuracy when the reading materials appeared on the screen. The reading materials were 120 common Chinese composite characters, each of them comprising phonetic and semantic radicals. The materials were categorized into three phonological conditions: regular-consistent (RC), regular-inconsistent (RiC), and irregular-inconsistent (iRiC). Notably, irregular-consistent (iRC) characters do not exist in Chinese. Similarly, based on semantic relationships, materials were classified as transparent or opaque. Therefore, the materials consisted of six types of characters (3 phonological conditions × 2 semantic conditions), each of them comprising 20 characters. An example character for each type is shown in Figure 1*B*. The word frequency of these characters was balanced across conditions. For the sake of simplicity, we did not consider the interaction mechanisms between the phonological and semantic conditions. Instead, we focused on the three individual sublexical effects, which were categorized as follows: consistency effects (40 RC vs 40 RiC), regularity effects (40 RiC vs 40 iRiC), and transparency effects (60 transparent vs 60 opaque). These effects were identified based on five combined character conditions: 40 RC, 40 RiC, 40 iRiC, 60 transparent, and 60 opaque characters. Thus, each analysis initially extracted the signals from each of the five conditions and then compared them to reveal each sublexical effect.

The experiment was conducted using Psychtoolbox (Brainard, 1997). For each trial, a cross-fixation point was initially displayed at the center of the screen for 0–1,000 ms, followed by a black screen for 2,000 ms, and then the presentation of a Chinese character for 1,000 ms. Participants were instructed to read the characters aloud as quickly and accurately as possible during the character presentation (Fig. 1*A*). The entire experimental task comprised six blocks, each consisting of 20 characters. In each block, the characters were selected pseudorandomly from the 120 Chinese characters. The selection ensured that each block had a balanced distribution of characters across the three phonological levels (regular-consistent, regular-inconsistent, irregular-inconsistent) and two semantic transparency levels (transparent, opaque). The characters within each block were presented in a randomized order to prevent order effects across participants. The entire experiment lasted ∼20 min.

### Behavioral data analysis

Recordings of each character were imported into Adobe Audition (2022) for manual marking of response time (RT) and participants’ pronunciation accuracy. Only the correct trials were used to calculate the average RT for each participant. A paired sample *t* test was used to examine RT and accuracy for the three sublexical effects.

### SEEG data acquisition

**Figure 2.**
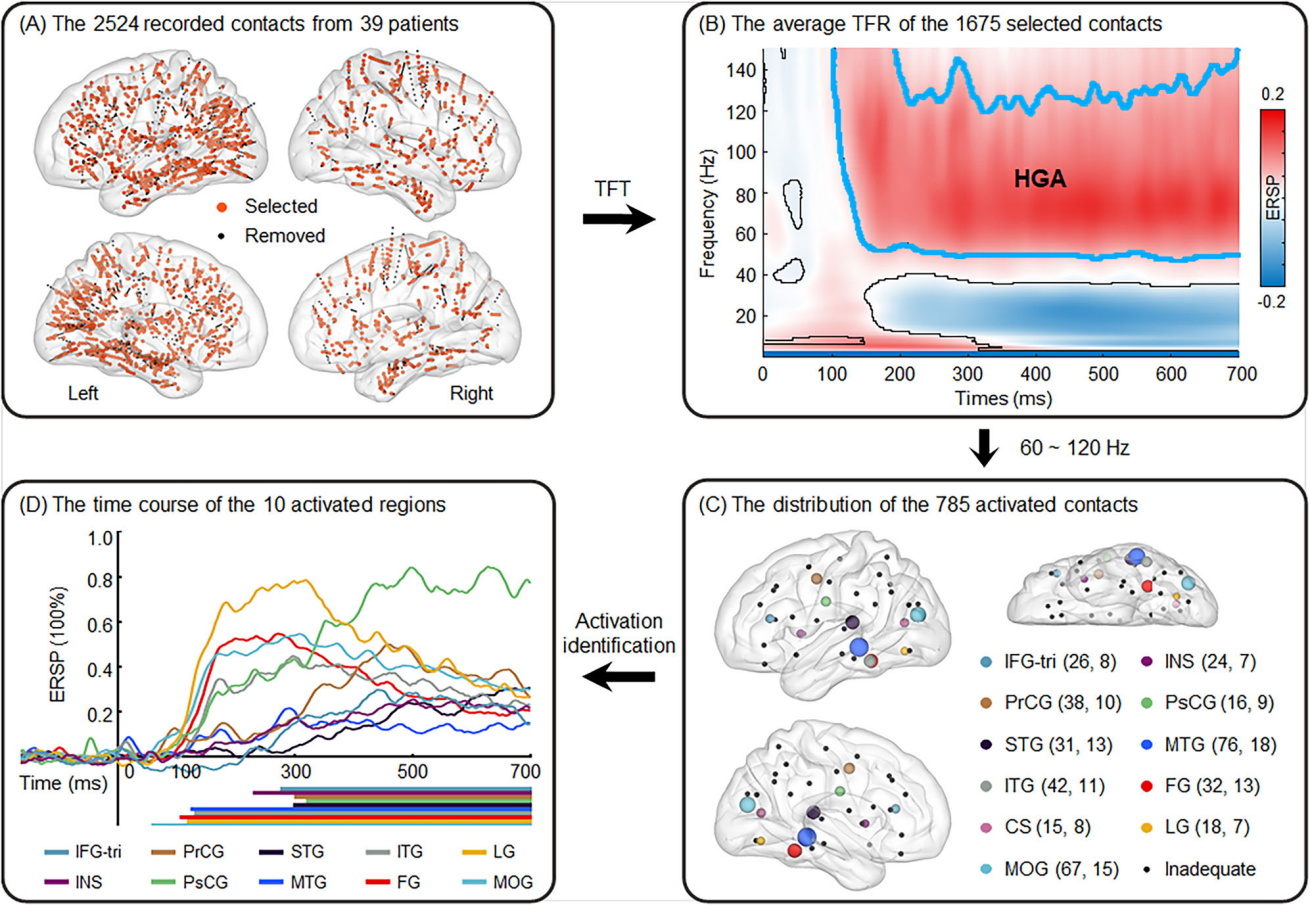
Contact selection and distribution among all the participants, along with overall and individualized activities for distinct brain regions. ***A***, A total of 2,524 contacts across the 39 participants were plotted on a standardized brain template. After removing 307 outliers and 542 contacts not in the gray matter (black small dots), we selected 1,675 contacts (orange dots) for subsequent analysis. ***B***, Grand average TFRs of the 1,675 selected contacts. The *x*-axis is time locked to stimulus onset, the *y*-axis represents the frequency band, and the contour line outlines the time window and frequency bands that were significantly different from the baseline (by FieldTrip, two-tailed, *p *< 0.05, Monte-Carlo 1000 randomizations). Consistent with previous studies, there was significant HGA (blue outline). ***C***, Out of the 1,675 contacts, 785 were responsive to the task in the HGA (60–120 Hz) and were distributed across different brain regions. The colored dots (11) highlight the effective regions meeting the further analysis criteria (with ≥15 responsive contacts from ≥7 participants, left hemisphere), with the dot size indicating the number of contacts in each region. The small black dots (26) indicate regions that inadequately met the criteria. The numbers in parentheses indicate the number of contacts (before the comma) and how many participants these contacts came from. ***D***, The ERSP time course for the identified activated regions (10) averaged across all epochs and all contacts. The *x*-axis is time locked to stimulus onset, the *y*-axis represents the percentage change in ERSP relative to baseline, and the colored lines below the axis mark time windows were significantly more active than the baseline (cluster-based permutation *t* test, 1,000 randomizations, one-tailed, *p *< 0.05, *FDR* corrected). TFT, time-frequency transformation; HGA, high gamma activity; TFR, time-frequency representation; ERSP, event-related spectral perturbation. IFG-tri, inferior frontal gyrus, triangular part; INS, insula; PrCG, precentral gyrus; PsCG, postcentral gyrus; STG, superior temporal gyrus; MTG, middle temporal gyrus; ITG, inferior temporal gyrus; FG, fusiform gyrus; CS, calcarine sulcus; LG, lingual gyrus; MOG, middle occipital gyrus. These brain regions are exclusively from the left hemisphere.

Thirty-nine participants were implanted with 401 electrodes. These electrodes comprised 5,703 contacts across all the participants (average 14.22 per electrode; SD = 2.78; range, 8–18). Contacts at electrode ends, near the seizure zone, or with high impedance (>15 kΩ) were excluded from recordings due to unstable signals. A total of 2,524 contacts were recorded (Table 1, Fig. 2*A*), with more in the left hemisphere (1,668) than in the right hemisphere (856). SEEG signals were obtained using the 64-channel EEG system by BrainAmp amplifiers (Brain Products). All channels were sampled at 5,000 Hz and referenced online to a scalp contact at the vertex.

**Table 1.** Background information of the 39 participants

Participant code	Gender	Age (years)	Education (years)	Handedness	Seizure zone	Number of contacts (recorded/implanted)
1CHY	Female	40	16	Right	Left anterior temporal lobe	64/114
2CGX	Male	15	8	Left	Right postcentral gyrus	64/122
3CJW	Male	26	16	Left	Left temporal lobe	64/221
4CJY	Female	13	9	Right	Right basal frontal lobe frontal, right insula	64/118
5CJY	Male	22	17	Left	Left temporal lobe	64/142
6GQZ	Male	29	N/D	Right	Left frontal lobe	62/154
7HS	Male	25	11	Right	Right temporal lobe, right insula	64/156
8JLF	Male	26	9	Right	Left superior temporal gyrus	63/176
9JP	Male	16	10	Right	Left central sulcus, left cingulum gyrus	64/153
10KJQ	Male	13	8	Right	Right frontal lobe	44/46
11LBX	Male	28	8	Right	Right basal frontal cortex	64/150
12LCJ	Male	23	11	Right	Left frontal lobe, left precentral gyrus	64/116
13LJ	Female	31	6	Right	Right anterior temporal lobe	64/152
14LJ	Male	18	12	Right	Left anterior temporal lobe	64/150
15LJ	Male	20	N/D	Right	Left calcarine sulcus, left cingulum gyrus	63/200
16LJH	Male	24	N/D	Right	Right postcentral gyrus	64/126
17LJQ	Male	25	9	Right	Right frontal lobe	64/132
18LL	Female	25	14	Right	Left anterior temporal lobe	64/164
19LR	Male	29	11	Right	Left occipital lobe	64/166
20LYP	Female	31	14	Right	Left insula, left temporal lobe	64/176
21LYT	Female	16	11	Right	Bilateral parieto-occipital sulcus	64/156
22LZH	Female	31	11	Right	Left middle frontal gyrus	64/96
23QK	Male	34	14	Right	Left middle temporal gyrus, left inferior parietal lobe	64/181
24SH	Male	29	14	Right	Left frontal lobe, left precentral gyrus	53/104
25SJX	Male	21	N/D	Right	Left hippocampus, left occipital lobe	128/172
26SXH	Male	28	9	Right	Left parietal lobe	64/165
27WGH	Female	43	14	Right	Right frontal pole, right insula	64/142
28WRC	Male	12	6	Right	Left prefrontal lobe, left insula	64/110
29WY	Female	17	11	Right	Right temporal lobe	64/120
30WY	Female	31	16	Right	Right insula	64/178
31WZD	Female	13	5	Right	Left occipital lobe	64/155
32WZT	Male	22	11	Right	Left temporo-parieto-occipital junction	64/220
33XB	Male	35	16	Right	Left occipito-temporal cortex	64/130
34XHY	Male	16	11	Right	Left frontal lobe	64/122
35XPZ	Male	12	6	Right	Right parietal lobe	64/68
36XXY	Male	18	12	Left	Right insula	63/140
37ZLX	Male	15	11	Right	Right temporal lobe	64/168
38ZSB	Female	38	9	Left	Left temporo-parieto-occipital junction	64/156
39ZXB	Male	23	15	Right	Left inferior frontal gyrus, left insula	64/186

Handedness was assessed by the Edinburgh Handedness Inventory (Oldfield, 1971). The seizure zone of the participants was identified using preoperative computed tomography (CT), magnetic resonance imaging (MRI), positron emission tomography (PET), and stereoelectroencephalography (SEEG) recordings. N/D, no data.

### Localizing the contacts

To localize these intracranial contacts, we registered structural MRI scans to computed tomography (CT) scans for each participant using the FieldTrip toolbox (Oostenveld et al., 2011). For group analysis across participants, contacts from each participant were projected onto a standard Montreal Neurological Institute (MNI) reference brain (Babajani-Feremi et al., 2016). Using BrainNet Viewer software (Xia et al., 2013), contacts from all the participants were superimposed on a brain surface template for visualization (Fig. 2*A*).

### Identifying the effective contacts

We preprocessed the SEEG signals using the EEGLAB toolbox (Delorme and Makeig, 2004). They were downsampled to 1,000 Hz, rereferenced off-line to the average of the contacts, and filtered with a bandpass filter (0.05–180 Hz) and two notch filters (49–51 and 99–101 Hz). Continuous SEEG signals were segmented into 3,000 ms epochs (−1,000 to 2,000 ms relative to the stimulus onset). Baseline correction was applied to data preceding the stimulus (1,000 ms before stimulus onset). Epochs were discarded if the pronunciation was incorrect, the pronunciation RT exceeded 3 SDs, or the SEEG had a drift exceeding 350 μV.

To obtain the time-frequency representation (TFR) of the SEEG signal, we convolved the remaining SEEG data with complex Morlet wavelets (1–150, 1 Hz intervals, seven cycles, −700 to 700 ms relative to the stimulus onset) using the FieldTrip toolbox. We averaged the TFR for each contact across trials. Contacts outside gray matter or with TFR exceeding 4 SDs were considered outliers and discarded.

### Identifying the responsive frequency and contacts

TFR differences between poststimulus (0–700 ms) and prestimulus (−700 to 0 ms) are shown in Figure 2*B*. To identify the most responsive frequency band, preprocessed SEEG data were time-frequency transformed (at 50–150, 5 Hz intervals). The transformation parameters were unchanged, except that the spectral powers were not averaged across trials. We averaged amplitudes for each contact across various ranges (e.g., 50–55, 50–60, 55–60, 50–65, … 145–150 Hz) to obtain the high gamma activity (HGA). A cluster-based permutation (1,000 randomizations) *t* test (Maris and Oostenveld, 2007) was used to compare HGA after stimuli with those in the baseline (−400 to −100 ms) across trials. A contact was considered responsive if it exhibited a significant increase (one-tailed, cluster level *p *< 0.05). The inspection procedure was repeated across all frequency ranges to determine the band with the greatest number of responsive contacts. The responsive contacts in this frequency band were considered final responsive contacts and included in subsequent analyses. Lateralization analysis was performed using a chi-square test to examine differences in the distribution of responsive and nonresponsive contacts between the left and right hemispheres.

### Identifying effective regions for further analysis

The responsive contact locations were determined using the Anatomical Automatic Labeling (AAL) template. Statistical tests were conducted in the effective brain regions with ≥15 responsive contacts from ≥7 participants (Fig. 2*C*) to ensure the representativeness of the results. Subsequent analyses focused on the left hemisphere because the right hemisphere had an inadequate number of implanted and responsive contacts to meet this standard and due to left-lateralized language processing (Shaywitz et al., 1995; Knecht et al., 2000; Ocklenburg et al., 2014; Dai et al., 2024). Details of the effective brain regions for subsequent analysis are provided in Table 2.

**Table 2.** Information on the effective brain regions for subsequent analysis

Brain regions	Abbr.	Number of contacts	Number of participants	Mean MNI		
				x	y	z
Inferior frontal gyrus, triangular part	IFG-tri	26	8	−43	30	14
Insula	INS	24	7	−38	8	3
Precentral gyrus	PrCG	37	10	−42	−4	44
Postcentral pyrus	PsCG	16	9	−56	−11	27
Superior temporal gyrus	STG	31	13	−55	−30	11
Middle temporal gyrus	MTG	76	18	−57	−35	−6
Inferior temporal gyrus	ITG	42	11	−52	−43	−17
Fusiform gyrus	FG	32	13	−32	−44	−17
Calcarine sulcus	CS	15	8	−17	−68	11
Lingual gyrus	LG	18	7	−24	−68	−9
Middle occipital gyrus	MOG	67	15	−35	−78	17

Number of contacts: the number of contacts contained within that brain region. The number of participants: how many participants these contacts come from. Mean MNI: the average Montreal Neurological Institute (MNI) template contact coordinates within that brain region. Abbr., abbreviation.

### Investigating activities and time courses in the effective regions

To investigate the activity and time course for each effective region, we averaged the HGA (60–120 Hz) of responsive contacts across trials and normalized as percentage changes relative to baseline power (−400 to −100 ms). This procedure yielded the event-related spectral perturbation (ERSP) for each contact. Similar to before, the cluster-based permutation (1,000 randomizations) *t* test was used to compare HGA after stimuli with those in the baseline (−400 to −100 ms) across participants. A region was considered responsive if it exhibited a significant increase in activity (one-tailed, cluster level *p *< 0.05), correction for multiple comparisons using the Benjamini–Hochberg *FDR* method (Benjamini and Hochberg, 1995).

### Investigating sublexical effects on activity in the activated regions

To investigate the sublexical effects on activity in the activated regions, we computed HGA for each condition—RC, RiC, iRiC, transparent, and opaque—to investigate the sublexical effects on activity (Fig. 1*B*). The cluster-based permutation *t* test (dependent sample, two-tailed, 1,000 randomizations) identified the consistency (RC vs RiC), regularity (RiC vs iRiC), and transparency (transparent vs opaque) effects in each activated brain region across participants. A region was considered to exhibit a consistency effect if it showed a significant difference between RC and RiC (*p *< 0.05, after *FDR* correction). The significance criterion for regularity and transparency effects was the same as that for consistency effect.

### Investigating connectivity among the regions exhibiting sublexical effects

To investigate whether sublexical processing occurs independently or collaboratively across brain regions, we utilized Granger causal analysis to examine the general connectivity among regions exhibiting sublexical effects (Granger, 1969; Stokes and Purdon, 2017). This method enabled us to evaluate directed information flow (Bressler and Seth, 2011; Yang et al., 2023). First, all preprocessed data underwent detrending, first-order differencing, and mean voltage subtraction to improve signal stationarity without considering experimental conditions. The traditional multivariate autoregressive (MVAR) model usually optimizes model order using criteria such as Akaike (Akaike, 1974) or Bayesian information (Schwarz, 1978). However, these criteria may not accurately estimate the model order for nonstationary time series (Havlicek et al., 2010). To prevent overfitting caused by excessive parameter estimation with high model order (Barnett and Seth, 2014), we selected an order of 10, as recommended by previous scalp and intracranial EEG studies (Brovelli et al., 2004; Gow et al., 2008; Narasimhan et al., 2020). Second, an MVAR model was used to fit the SEEG signals from all the region contacts. The model operated in continuous, overlapping 200 ms time windows with a 10 ms step from −200 to 700 ms related to the stimulus onset. Third, coefficient matrices from the MVAR model underwent a Fourier transformation to obtain their frequency-domain representation. Finally, the Granger–Geweke estimator was used to compute the Granger causal value (GCV) within 60–120 Hz (Geweke, 1982).

Due to the connectivity analysis being limited to participants with contacts in two regions forming pathways, only specific participants were included. We applied nonparametric statistics to the average GCV across contacts within each participant. If the GCV was higher (one-tailed, 1,000 randomizations, *FDR* corrected across the time, *p *< 0.05) than the random distribution (Perrone-Bertolotti et al., 2012; Si et al., 2017; Liu et al., 2021; Li et al., 2022), we considered that there was connectivity within this participant. To minimize false-positive results, we inferred connectivity only when more than half of the participants exhibited significant connectivity (Liu et al., 2021).

### Investigating sublexical effects on connectivity between connected regions

The above Granger causality analysis elucidates connectivity within sublexical processing brain regions. However, it remains unclear whether the information flow between these regions is linked to sublexical information. To address this issue, we investigated the sublexical effects on connectivity. Specifically, we calculated the GCV for each contrast level (RC, RiC, iRiC, transparent, and opaque) and compared the connectivity differences between them. Similar to the HGA comparisons, the cluster-based permutation *t* test identified the effects of consistency, regularity, and transparency. Statistical tests were limited to brain regions with ≥7 participants to ensure representativeness and sufficient permutations. The significance criterion for the GCV difference between conditions matched that applied to the HGA difference.

### Data availability

The preprocessed SEEG data and processing scripts are available at https://osf.io/mkz2r/.

## Results

### Behavioral performance

The reading RTs of 39 participants in the task are shown in Figure 1*C*. At the phonological level, the RC RT (708 ± 16 ms, mean ± standard deviation) was significantly shorter than the RiC RT (722 ± 15 ms; *t*_(38) _= 18.12; *p *< 0.001; Cohen's *d *= 2.90); the RiC RT was significantly shorter than the iRiC RT (732 ± 25 ms; *t*_(38) _= 4.66; *p *< 0.001; Cohen's *d *= 0.75). At the semantic level, the transparent RT (715 ± 19 ms) was significantly shorter than the opaque RT (727 ± 18 ms; *t*_(38) _= 16.33; *p *< 0.001; Cohen's *d *= 2.62). There was no significant difference in accuracy between the three levels (RC: 0.95 ± 0.06; RiC: 0.95 ± 0.04; iRiC: 0.94 ± 0.05) of phonological factors (*t*s_(38) _< 1.53; *p*s > 0.14; Cohen's *d *< 0.25) or the two levels (transparent, 0.94 ± 0.04; opaque, 0.96 ± 0.05) of semantic factors (*t*_(38) _= 1.93; *p *= 0.06; Cohen's *d *= 0.31). The behavioral results suggest that there were sublexical effects, indicating the processing of sublexical phonological and semantic information during the character reading task.

### The overall activity and time course during the task

We recorded 2,524 contacts from the 39 participants. After removing 307 outliers and 542 nongray matter contacts, 1,675 contacts remained (1,178 left vs 497 right; Fig. 2*A*). The inspection procedure showed that the frequency band with the highest number (785) of responsive contacts for the HGA was 60–120 Hz. Lateralization analysis revealed that the responsive contacts (582 left vs 203 right) were more located in the left hemisphere than the nonresponsive contacts (596 left vs 294 right; *χ*^2 ^= 9.95; *p *= 0.002; *Φ* = 0.08). We identified 11 effective left brain regions (with ≥15 responsive contacts from ≥7 participants) for subsequent analysis (Table 2). They were inferior frontal gyrus, triangular part (IFG-tri), insula (INS), precentral gyrus (PrCG), postcentral gyrus (PsCG), superior temporal gyrus (STG), middle temporal gyrus (MTG), inferior temporal gyrus (ITG), fusiform gyrus (FG), calcarine sulcus (CS), lingual gyrus (LG), and middle occipital gyrus (MOG).

During the task, significant activity was observed in 10 brain regions, without the CS (Fig. 2*D*). Based on activity latency, these regions can be divided into two categories: lower-level language processing regions with shorter latency and higher-level language processing regions with longer latency. The former, beginning activity ∼100 ms poststimulus, included the MTG, ITG, FG, LG, and MOG. In contrast, the latter, activating ∼300 ms poststimulus, encompassed the IFG-tri, INS, PrCG, PsCG, and STG. These results indicate that the reading task involved activities in multiple brain regions. They were activated at different times, which may reflect their distinct roles in reading tasks.

### The sublexical effects on activity in the activated regions

**Figure 3.**
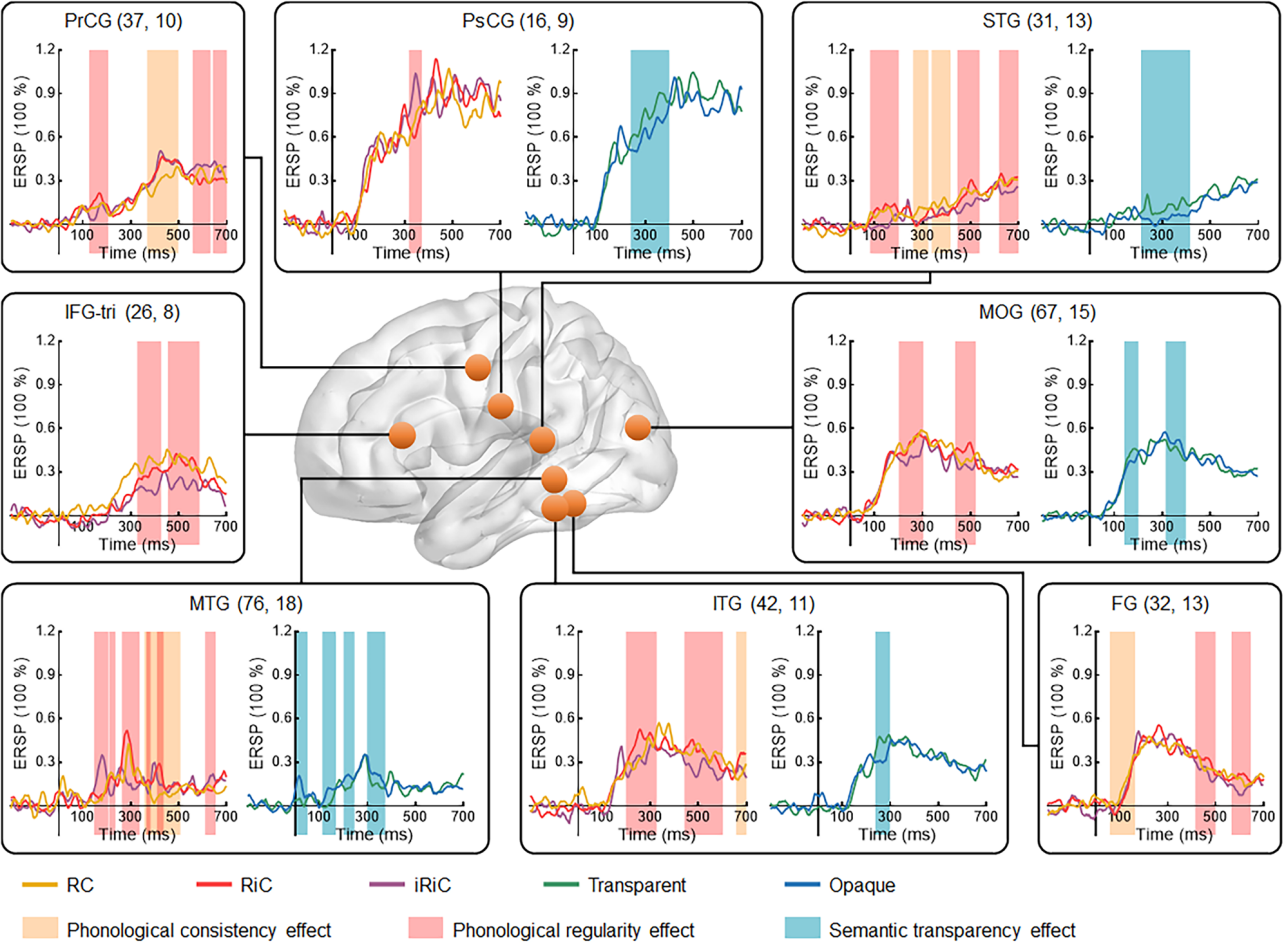
Sublexical effects on HGA in activated brain regions. The *x*-axis is time locked to stimulus onset, the *y*-axis represents the percentage change in ERSP relative to baseline, and the colored rectangular bars indicate significant time windows for the phonological consistency effect (RC vs RiC, yellow), phonological regularity effect (RiC vs iRiC, red), and semantic transparency effect (transparent vs opaque, blue). Refer to Figures 1 and 2 for the full names of the conditions and brain regions.

Figure 3 illustrates significant phonological consistency effects in five regions: PrCG (368–498 ms), STG (260–416 ms), MTG (358–508 ms), ITG (658–700 ms), and FG (58–164 ms). There were significant phonological regularity effects in eight regions: IFG-tri (328–588 ms), PrCG (126–206, 558–700 ms), PsCG (318–372 ms), STG (80–200, 444–538, 618–700 ms), MTG (148–438, 612–656 ms), ITG (198–326, 442–602 ms), FG (414–500, 566–646 ms), and MOG (202–304, 436–522 ms). Additionally, significant semantic transparency effect existed in five regions: PsCG (238–398 ms), STG (212–416 ms), MTG (0–374 ms), ITG (238–298 ms), and MOG (142–200, 314–398 ms). Most regions exhibited both phonological and semantic sublexical effects, except for the IFG-tri and FG, which showed only phonological sublexical effect, with no region exhibiting solely semantic effects. Similarly, five regions exhibited both phonological consistency and regularity sublexical effects, except for the IFG-tri, PsCG, and MOG, which only showed regularity sublexical effect, with no region showing exclusively consistency effects. These findings suggested that sublexical effects were distributed across the frontal, temporal, and occipital networks. These findings suggest that the brain processed more phonological information, especially when the sublexical pronunciation differs from the whole character, rather than semantic information. This may be related to the oral reading task in this study.

### The connectivity among the regions exhibiting sublexical effects

**Figure 4.**
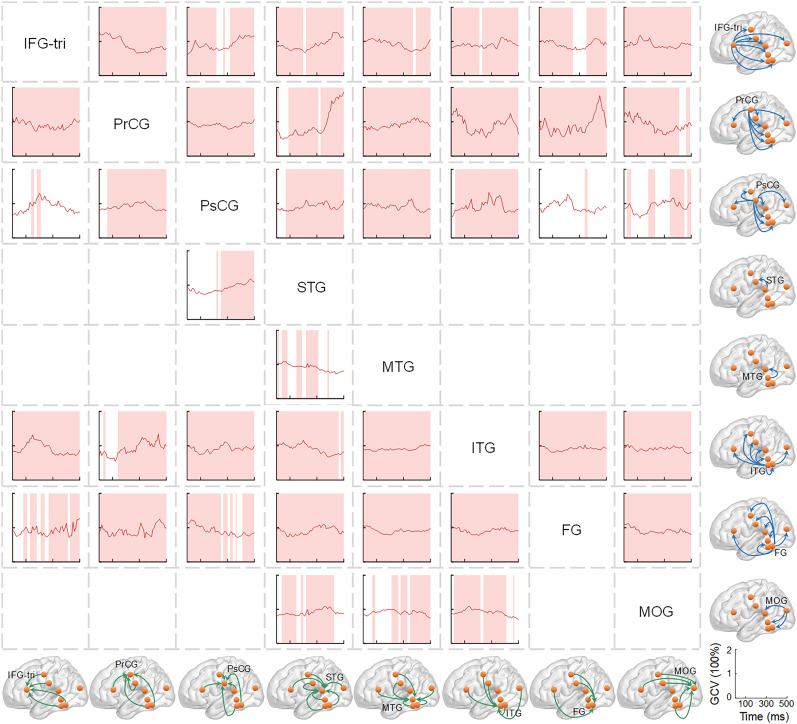
The connectivity results for each pathway between brain regions exhibiting sublexical effects. Across the entire image, the subdiagrams listed in columns represent information reception from others to them (green arrows), while those listed in rows represent information transmission from them to others (blue arrows). In each subdiagram, the *x*-axis is time locked to stimulus onset, the *y*-axis is the percentage change in GCV relative to baseline, and the rectangular bars indicate time windows where more than half of the participants exhibited a significantly higher GCV than random distribution. Except for the diagonal subdiagram, areas without GCV results suggest no significant connectivity between the region pairs. GCV, Granger causal value. Refer to Figure 2 for the full names of the brain regions.

The Granger causality analysis revealed extensive connectivity among regions exhibiting sublexical effects (Fig. 4). The IFG-tri, PrCG, PsCG, ITG, and FG transmitted information to all other sublexical processing regions. In contrast, the STG and MTG rarely transmitted information to other regions. For example, the STG and MTG primarily received information from the IFG-tri, PrCG, and PsCG, with minimal transmission back, except from the STG to PsCG. Similarly, in the temporal lobe, information flowed from the FG and ITG to the STG and MTG, but not vice versa, showing a bottom-up characteristic. These results also suggest that sublexical processing involves coordination among multiple regions rather than independent activation.

### The sublexical effects on connectivity between the connected regions

**Figure 5.**
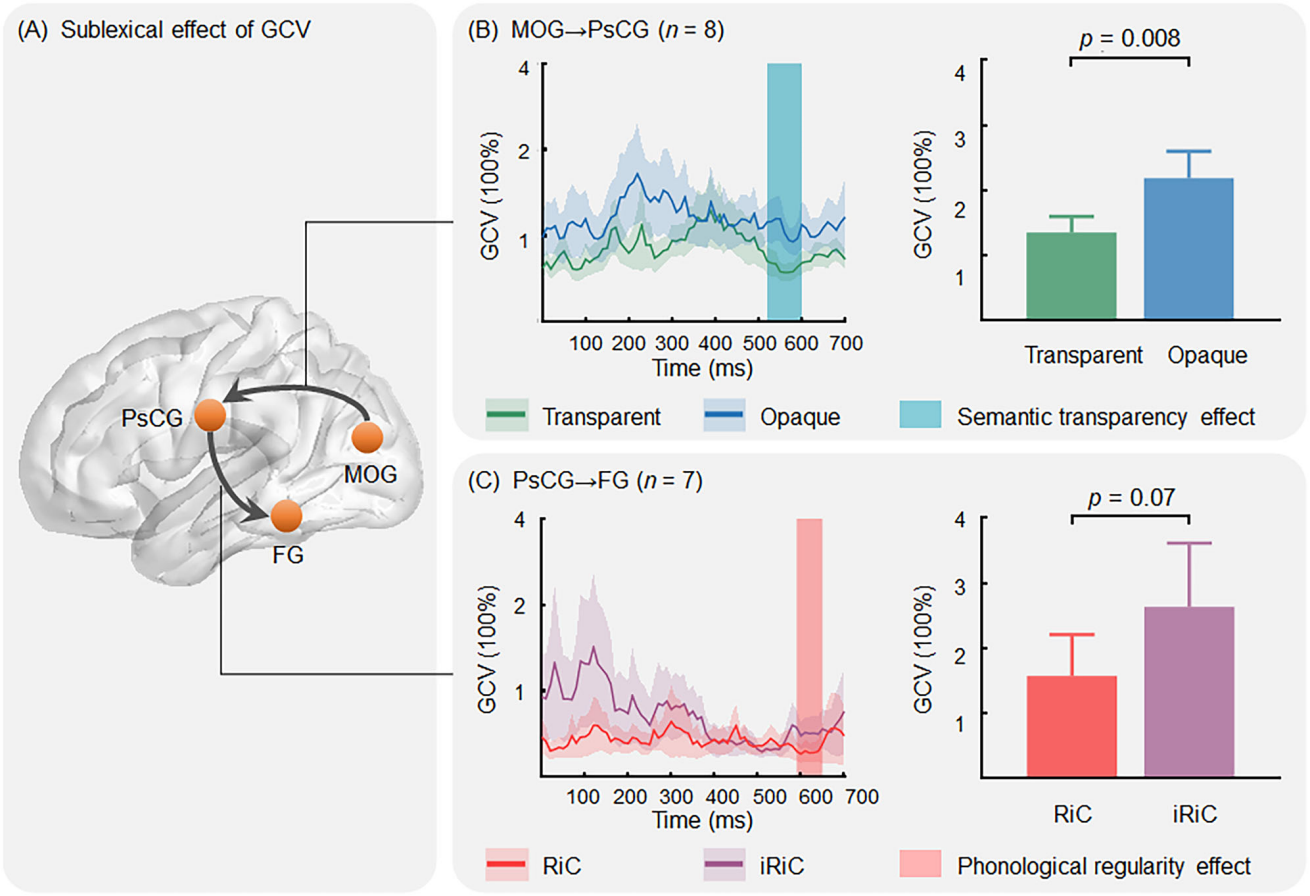
The sublexical effects of GCV. ***A***, Two pathways (from MOG to PsCG and from PsCG to FG) exhibited sublexical effects. ***B***, GCV results for transparent and opaque conditions from the MOG to the PsCG (left) as a function of time. The *x*-axis is time locked to stimulus onset, the *y*-axis represents the GCV, and the rectangular bar highlights a significant time window for the semantic transparency effect (transparent vs opaque). The right side displays the mean GCV within the significant time window between the two conditions. ***C***, GCV results for RiC and iRiC conditions from the PsCG to the FG (left) as a function of time and the mean GCV within the significant time window between the two conditions (right). The shading around these lines represents the standard error. Refer to Figures 1 and 2 for the full names of the conditions and brain regions.

The above results elucidated connectivity within sublexical processing brain regions. However, it is unclear whether the connectivity was related to sublexical processing. Examining differences in connectivity between conditions can illustrate this matter. Analysis of GCV differences revealed two sublexical effects (Fig. 5). A semantic transparency effect was observed in the pathways from the MOG to PsCG (520–600 ms), while a phonological regularity effect was observed in the pathways from the PsCG to FG (590–650 ms). These results suggest that the changes in sublexical information influence the information flow between specific regions emphasizing the involvement of these brain regions and their information exchange in sublexical processing.

## Discussion

We recruited 39 epilepsy patients to complete a Chinese character reading task and examined the neural mechanisms of sublexical processing using SEEG implanted in their brains. Our observations included the following: (1) 31.10% (785/2,524) of contacts responded to the tasks; 10 brain regions (IFG-tri, INS, PrCG, PsCG, STG, MTG, ITG, FG, LG, and MOG) were activated during the tasks; (2) sublexical processing involved the IFG-tri, PrCG, PsCG, STG, MTG, ITG, FG, and MOG; (3) connections were observed between these sublexical processing regions; and (4) connections from the MOG to PsCG and from the PsCG to FG showed sublexical effects.

### Regions engaged in sublexical processing

Similar to previous neuroimaging studies exploring sublexical effects through phonological/orthographic manipulations (Okada and Hickok, 2006; Vinckier et al., 2007; Vaden et al., 2011; Gow and Nied 2014; Gow and Olson 2015; Avcu et al., 2023; Regev et al., 2024), our study found sublexical processing in the temporal lobe (Paulesu et al., 1993; Okada and Hickok, 2006; DeWitt and Rauschecker, 2012; Gow and Olson, 2015; Lopopolo et al., 2017; Scott and Perrachione 2019; Regev et al., 2024) and the inferior frontal cortex (Paulesu et al., 1993; Démonet et al., 1994; Poldrack et al., 1999; Myers et al., 2009; Vaden et al., 2011; Okada et al., 2018; Xie and Myers, 2018; Regev et al., 2024) using SEEG. Furthermore, our findings of sublexical phonological effects in the frontotemporal network were also consistent with studies on Chinese sublexical phonological processing (Tan et al., 2001; Yang et al., 2011). Moreover, some of these regions (IFG-tri, STG, ITG, and FG) showed smaller amplitudes for conflicting characters and larger amplitudes for nonconflicting characters (RC > RiC or RiC > iRiC), which may suggest their involvement in sublexical phonological information retrieval (Mano et al., 2013). When reading irregular/inconsistent characters, sublexical pronunciation disrupted the whole character pronunciation. These irregular/inconsistent patterns match the stored sublexical pronunciation to a lesser degree, and sublexical extraction was suppressed. Additionally, we found that PrCG and PsCG were involved in sublexical phonological processing and exhibited a contrasting pattern: conflicting characters induced increased activity, while nonconflicting characters induced decreased activity (RC < RiC or RiC < iRiC). It may imply that PrCG and PsCG contribute to a phonological control process (Bouchard and Chang, 2014). When a discrepancy arises between sublexical and lexical pronunciations, activity in these regions increases to suppress sublexical pronunciation, possibly due to heightened sensorimotor effort.

Previous research highlighted the frontal cortex's significant role in lexical-semantic systems (Corrivetti et al., 2019; Woolnough et al., 2022; Chiou et al., 2023; Murphy et al., 2023), with the left IFG being causally involved in selective semantic retrieval (Zhao et al., 2021). In our study, regions exhibiting sublexical semantic effects were mainly clustered in the temporal lobe, PsCG, and MOG rather than the frontal lobe. This phenomenon might result from the oral reading task employed in our study, where participants focused on speech retrieval without significant demand for semantic retrieval. This finding implies that frontal cortex involvement in semantic access may not be entirely automatic but requires heightened active cognitive engagement (Roskies et al., 2001; Huang et al., 2012). The finding that passive semantic extraction weakened semantic representation in the inferior frontal gyrus compared with active semantic extraction could support this perspective (Liuzzi et al., 2021).

### Connectivity between sublexical processing regions

The Granger causal analysis revealed extensive information flow (connectivity) within the sublexical processing network. Liu et al. (2021) showed information feedback from higher-level linguistic regions (e.g., the IFG-tri, INS, and PsCG) to lower-level linguistic regions (e.g., the ITG, FG, and MOG). However, the study focused on information exchange among brain regions involved in whole word processing. The specific roles of sublexical processing networks during word recognition remain unclear. The current study concentrated on sublexical processing brain regions, revealing their bidirectional information exchange dynamics. These results emphasize that sublexical processing may not be an isolated process conducted by distinct brain regions but may involve coordinated interactions across numerous large-scale brain regions (Thiebaut de Schotten and Forkel, 2022).

Interestingly, we observed sublexical effects on connectivity in the pathways from the MOG to the PsCG and from the PsCG to the FG. Both of them exhibited greater connectivity in the conflicting condition than in the nonconflicting condition (iRiC > RiC or Opaque > Transparent). The results obtained from HGA further confirm the important role of PsCG in conflict representation between lexical and sublexical. When a discrepancy arises between sublexical and lexical pronunciations/meanings, PsCG receives information from other brain regions to control sensorimotor, while transmitting this conflicting information to other brain regions to reduce the sublexical representation and processing (Jobard et al., 2003; Borowsky et al., 2006).

### Modulation from higher- to lower-level linguistic regions during word reading

Our findings reveal that word processing involves both feedforward and feedback networks between linguistic regions. Feedforward networks transmit information from higher-level regions like IFG-tri, PrCG, and PsCG to other sublexical processing regions (Liu et al., 2021). Conversely, feedback networks convey information from lower-level regions like ITG and FG to all the other sublexical processing regions. Interestingly, while STG and MTG are central language regions expected to transmit information to motor regions, they transmit less information overall. We propose that this transmission occurs through intermediate steps, such as MTG→STG→PsCG and STG→PsCG→IFG. The observed information flow from MTG to STG, STG to PsCG, and PsCG to IFG supports this hypothesis.

Another intriguing observation is that the lower-level linguistic processing regions within the temporal lobe (ITG and FG), which specializes in visual word form processing (Cohen et al., 2000; Dehaene and Cohen, 2011; Lerma-Usabiaga et al., 2018; Liu et al., 2021; Woolnough et al., 2021), exhibited sublexical effects. This finding suggested that they not only process lower-level word form but also sublexical information (Gold et al., 2006; Fischer-Baum et al., 2017; Zhao et al., 2017). This phenomenon may be attributed to feedback from other sublexical processing regions, as evidenced by earlier sublexical effects in the PrCG and PsCG and the connectivity between them. Previous research on structural and functional connectivity between the ventral occipital temporal cortex and other sublexical-related brain regions could provide support for this perspective (Saygin et al., 2016; Stevens et al., 2017; Moulton et al., 2019).

### Limitations

The current study has limitations that should be considered. (1) Unequal stimulus numbers across sublexical effects (consistency effect, 40 vs 40 trials; regularity effect, 40 vs 40 trials; and transparency effect, 60 vs 60 trials) might lead to differences in observed effect size for these sublexical effects. (2) The primary task employed in this study was a reading task, which could weaken sublexical semantic effects. (3) All participants in this study were epilepsy patients, and electrode implantation was determined by clinical necessity. Therefore, adequate contact across brain regions cannot be guaranteed, which may limit the detection of sublexical effects in specific brain regions within a more representative population.

### Conclusion

Our analysis of SEEG signals from epilepsy patients revealed the dynamics and connectivity within the sublexical processing neural network. It engages the IFG-tri, sensorimotor cortex, temporal lobe, and MOG, with intricate information interactions between them. Furthermore, characters with different radical types induced variable connectivity. This study provides valuable insights into the neural mechanisms of sublexical processing.
